# A NEW LOOK AT AN OLD PROBLEM: AN EDITOR’S PERSPECTIVE ON BARRIERS TO GROWING AN AGE-SAVVY WORKFORCE

**DOI:** 10.1093/geroni/igae098.1036

**Published:** 2024-12-31

**Authors:** Rona Karasik

**Affiliations:** Saint Cloud State University, Saint Cloud, Minnesota, United States

## Abstract

The future sustainability of gerontology and geriatrics programs has been the subject of concern well before Gerontology & Geriatrics Education (G&GE) was established in 1980, and remains a persistent theme in the journal today. Dwindling financial support for gerontology programs has also been an issue since before the journal’s inception (Wittels, 1981), as was the much anticipated and now present shift in age demographics and its accompanying need to educate a sufficient, age-savvy workforce. In other words, none of these problems are new. Despite innovative curricula and the burgeoning number of older adults, the question remains steadfast: “Why don’t more students choose to go into gerontology or geriatrics?” Moreover, what can be done to attract more students as the need for aging professionals continues to increase and enrollments in gerontology and geriatrics programs decline? The current presentation explores trends in the work submitted to Gerontology & Geriatrics Education, the Academy for Gerontology in Higher Education (AGHE)’s official journal, that seek to: (a) identify reasons why students do not choose gerontology (e.g., ageism, prestige, pay differentials); (b) evaluate novel strategies to address this disinterest (e.g., intergenerational programs, Ageism First Aid); and (c) endeavor to secure the future for gerontology and geriatrics educational programs (e.g., outreach to general education, community partnerships) amid the context of sweeping budget cuts throughout higher education.

